# Challenges for Emergent Combined Cesarean Delivery and Type A Aortic Dissection Repair Including Bleeding Management in the Setting of Full Heparinization: A Case Report

**DOI:** 10.7759/cureus.30647

**Published:** 2022-10-24

**Authors:** Cory Y Lin, Chelsea L Stiles, Sudhakar Subramani, Matthew J Maxwell, Dionne F Peacher, Sharon B Larson, Satoshi Hanada

**Affiliations:** 1 Anesthesia, University of Iowa Hospitals and Clinics, Iowa City, USA; 2 Anesthesia, Iowa Methodist Medical Center, West Des Moines, USA; 3 Anesthesia, Houston Methodist Hospital, Houston, USA; 4 Cardiothoracic Surgery, University of Iowa Hospitals and Clinics, Iowa City, USA

**Keywords:** cesarean delivery, anesthesia, aortic dissection repair, pregnancy, aortic dissection

## Abstract

Type A aortic dissection is rare in young females; however, it is associated with a high mortality rate. This case report describes a 30-year-old female at 38 weeks of gestation who presented with acute onset chest pain and hypotension responsive to intravenous fluid therapy. Transthoracic echocardiogram and chest computed tomography angiography confirmed a type A aortic dissection. The patient was transported urgently to the operating room for a Cesarean section and aortic dissection repair. Following induction of general anesthesia, the baby was delivered, oxytocin infusion was started, and a Bakri balloon was placed in the uterus. On cardiopulmonary bypass with circulatory arrest, the ascending aorta and aortic valve were repaired. Multiple uterotonic agents were required intraoperatively to manage persistent uterine bleeding in the setting of full heparinization. Both mother and baby survived without major complications. Preoperative management should focus on maternal hemodynamic control while completing a diagnostic evaluation. Intraoperative considerations include minimizing fetal exposure to medication, maintaining hemodynamic stability, and managing intraoperative blood loss in the setting of full anticoagulation.

## Introduction

Type A aortic dissection is a rare pathology in young females; however, when present during pregnancy, it can be devastating to both the mother and the fetus [1]. Pregnancy contributes to physiologic changes, including greater cardiovascular stress and hyperdynamic circulation that increases the risk of dissection. This risk is increased even further in patients with genetic connective tissue disorders or pre-existing aortopathy [2]. Each step of management in these patients requires risk and benefit analysis for both the mother and fetus.

## Case presentation

**Figure 1:**
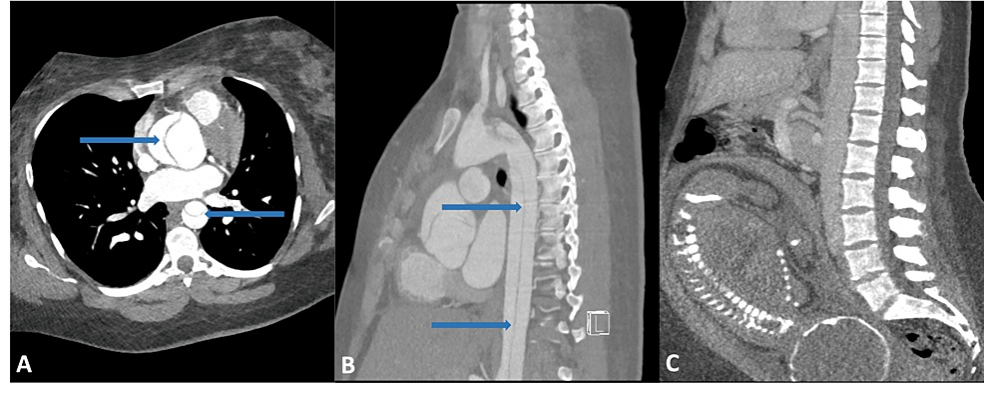
Computed tomography angiogram of the chest and abdomen (A) Axial image shows type A aortic dissection with a dissection flap (arrows) originating distal to the aortic root. (B) Sagittal image shows the dissection flap (arrows) continuing distally into the abdominal aorta. (C) Sagittal image shows the fetus in a cephalic position within the uterine cavity.

A previously healthy 30-year-old Caucasian female gravida 4 para 1 (height 152 cm, weight 100 kg, body mass index 36 kg/m^2^) at 38 weeks and four days of gestation with a significant family history of aortic dissection and aortic aneurysm rupture presented with acute onset right upper chest pain radiating to her low back associated with vision changes and paresthesia to her hands. Known aortic root dilation without aortic dissection was present during her previous pregnancy; induction of labor was performed and there were no complications during delivery. During this present case, the patient was transferred to our academic center from an outside hospital that had minimal resources for cardiothoracic intervention should a dissection be imminent. There, she had been hypotensive at 60/30 mmHg but rapidly improved following a bolus of 2,000 mL of crystalloid. On arrival, she reported pain in her low back consistent with contractions, noted positive fetal movement, and denied vaginal bleeding or loss of fluid. Category 1 fetal heart tracing was noted, and ultrasound showed the fetus in cephalic presentation. Dizziness and lightheadedness had resolved with the 2,000 mL bolus of fluids at the outside hospital. Chest pain had also improved from the time she initially presented to the outside hospital. Bedside transthoracic echocardiogram (TTE) was concerning for a large dissection flap in the aortic root, a dilated ascending aorta at 3.9 cm, and moderate aortic insufficiency. A right radial arterial line was placed using the sterile technique for close hemodynamic monitoring and the patient was started on nicardipine infusion with a target mean arterial pressure (MAP) of 60 mmHg and esmolol infusion with a target heart rate of 60-70 beats per minute. Emergent computed tomography angiography (CTA) of the chest, abdomen, and pelvis confirmed a type A aortic dissection with a fenestrated dissection flap originating distal to the aortic root (no coronary involvement) and extending through the aortic arch into the origins of the innominate artery, left common carotid artery, and left subclavian artery (Figures 1A-1C). The flap continued distally into the abdominal aorta to the level of the left renal artery. No pericardial effusion was found on either TTE or CTA.

After a multidisciplinary discussion between anesthesia, cardiothoracic surgery, obstetrics, and neonatology, a decision was made to proceed with an emergent Cesarean section followed immediately by aortic dissection repair. The patient consented to these interventions and was informed and understanding of all the potential complications and risks, including bleeding, blood transfusions, emergent hysterectomy, and death. She was transported to the operating room where invasive lines were placed under local anesthesia, including a large-bore peripheral intravenous (IV) catheter, a left brachial arterial line, a multi-lumen central venous catheter in the right internal jugular vein, and a pulmonary artery catheter. Fetal heart rate was continuously monitored in the operating room by the obstetric team. The patient was prepped and draped for both Cesarean section and cardiothoracic surgery. The neonatal intensive care unit (ICU) team was in the operating room and prepared to receive the infant. The patient remained hemodynamically stable on induction with propofol IV 150 mg, succinylcholine IV 140 mg, and remifentanil IV infusion at 0.2 mcg/kg/min. Anesthesia was maintained with remifentanil IV infusion at 0.05-0.2 mcg/kg/min and isoflurane inhalational MAC 0.5. No other anesthetic agents or opioids were used until after the baby was delivered. A transesophageal echocardiogram (TEE) was performed immediately following intubation, which confirmed the diagnosis of a type A aortic dissection with moderate aortic valve insufficiency. TEE also showed normal biventricular systolic function without wall motion abnormalities. No pericardial effusion was present. The baby was delivered within three minutes of anesthesia induction with Apgar scores of five and eight at one minute and five minutes, respectively. Immediately after delivery, an oxytocin IV infusion was started at 300 units/hour; however, due to persistent uterine atony, the infusion was increased to oxytocin IV at 600 units/hour and the patient received carboprost tromethamine (Hemabate) intramuscular (IM) 250 mcg with improvement in tone. The patient remained hemodynamically stable during the Cesarean section. The obstetric team reported adequate hemostasis and proceeded with the placement of a Bakri balloon in an effort to tamponade uterine bleeding during cardiopulmonary bypass (CPB).

After the closure of the Cesarean section incision, the cardiothoracic surgery team proceeded with the placement of CPB cannulas, including an arterial cannula in the right axillary artery before sternotomy and a venous cannula in the right atrium after sternotomy. The patient was fully heparinized and an activated clotting time (ACT) greater than 400 seconds was maintained during CPB. The patient was actively cooled to 25 degrees centigrade (monitored by both bladder and nasopharyngeal temperature probes) prior to circulatory arrest. The cardiothoracic surgery team performed an ascending aorta graft repair and aortic valve repair via commissural resuspension. Throughout the CPB period, persistent uterine bleeding, totaling 2,000 mL, was noted throughout the case. In an effort to decrease uterine blood loss, the patient received methylergonovine (Methergine) IM 0.2 mg and misoprostol (Cytotec) per rectum 600 mcg without significant improvement. Aminocaproic acid was also infused (1 gram per hour followed by a 5-gram bolus) throughout the case as part of the institutional protocol for cardiac surgery. The obstetric team periodically evaluated uterine tone and the position of the Bakri balloon during the CPB period. A residual arch tear into the descending aorta was identified by the surgical team; however, given persistent uterine bleeding, the decision was made to leave the residual arch dissection unrepaired. Total bypass time was 238 minutes, cross-clamp time was 174 minutes, and circulatory arrest time was 48 minutes with antegrade cerebral perfusion. The patient received an initial protamine IV 300 mg; however, due to persistent uterine bleeding, an additional protamine IV 90 mg was administered in intermittent boluses. The patient briefly required norepinephrine and vasopressin infusions while being weaned from CPB. The estimated blood loss was 1,000 mL during the Cesarean section and 2,800 mL during the aortic dissection repair, which included 2,000 mL of uterine blood loss during the CPB period. The patient received a total of six units (1,950 mL) of packed red blood cells, four units (1,162 mL) of platelets, two units (200 mL) of cryoprecipitate, and three units (950 mL) of fresh frozen plasma. The results of the heparinase thromboelastography (hTEG) obtained intraoperatively were within normal limits. The patient was transported to the cardiovascular ICU in stable condition on norepinephrine infusions, and she was extubated on postoperative day 1. The patient was neurologically intact. Her postoperative course was complicated by acute kidney injury, with a maximum serum creatinine level of 1.5 mg/dL, which resolved with supportive therapy. The patient and her baby were discharged home on postoperative day 6. The patient followed up with vascular surgery postoperatively to monitor her residual aortic arch dissection.

## Discussion

Acute type A aortic dissection is rare in young females; however, it is associated with a high mortality rate which increases by 1% every hour during the first 24 hours [3]. It is estimated by case reports that 50% of aortic dissections in females younger than 40 years of age are associated with pregnancy, primarily occurring during the third trimester or immediate postpartum period when hemodynamic stress is at its peak [4]. During pregnancy, the hormonal effects of both estrogen and progesterone alter the elastic fibers in the aorta [5]. The combination of these hormonal changes with a pre-existing aortic pathology, such as Marfan syndrome or a bicuspid aortic valve, is associated with an increased risk of aortic dissection [5]. Our patient had a significant family history of aortic aneurysm rupture and aortic dissection, suggesting a genetic predisposition to aortic pathology. The patient had previously undergone genetic evaluation; however, no specific syndrome or polymorphism was identified. She was under close surveillance for aortic pathology throughout her pregnancy with serial echocardiograms.

Preoperative management focuses on minimizing hypertension and tachycardia to decrease the propagation of the dissection and reduce the risk of rupture without compromising the fetal outcome. However, currently, there are no set guidelines for the hemodynamic management of pregnant patients with acute aortic dissection. The American College of Obstetricians and Gynecologists (ACOG) has published guidelines for the use of antihypertensive agents in the setting of severe hypertension in pregnant patients. These guidelines list IV labetalol and IV hydralazine as first-line therapies [6]. These medications have been used in pregnant patients for many years and have well-known side effect profiles, which include fetal bradycardia and maternal bronchospasm with IV labetalol, and maternal headaches and tachycardia with IV hydralazine [6-8]. If these agents fail to improve maternal hemodynamics, ACOG recommends the use of nicardipine or esmolol as second-line agents [6]. Nicardipine infusions can result in tachycardia, while esmolol crosses the placenta and can result in fetal bradycardia and persistent fetal beta-blockade [8]. Sodium nitroprusside is considered a third-line agent, should the above therapies fail, due to the risk of maternal and fetal cyanide and thiocyanate toxicity [6]. In a setting of type A aortic dissection, the patient can become acutely hemodynamically unstable if the dissection progresses or if the aorta ruptures. For that reason, medications with short onset and offset are preferred, making labetalol and hydralazine less ideal compared to other agents.

The anesthetic approach to pregnant patients with acute type A aortic dissection is a complex. The benefits of neuraxial anesthesia include minimizing the stress response to surgical incision, avoiding fetal exposure to general anesthesia, and allowing the mother to witness the birth of her child prior to undergoing major surgery. However, placing the patient in the lateral or sitting position to perform neuraxial anesthesia could risk further progression of dissection, even though little to no evidence exists to support this. Neuraxial anesthesia may also worsen hypotension in patients with normal or low blood pressure. Additionally, there is a concern for increased risks of bleeding and hematoma formation with neuraxial anesthesia in the setting of high-dose intraoperative heparin required during CPB [9]. In contrast to neuraxial anesthesia, general anesthesia provides a secure airway in a patient at high risk of aspiration (being a full-term pregnant patient undergoing emergency surgery) who can quickly become hemodynamically unstable. General anesthesia also allows the provider to place a TEE probe after intubation to obtain baseline images and monitor the progression of a dissection throughout Cesarean section.

Given the stability of our patient, we placed invasive monitors under local anesthesia, including a left brachial arterial line and a pulmonary artery catheter, to minimize fetal exposure to general anesthesia. Our goals on induction included avoiding propagation of the aortic dissection and maintaining adequate fetal perfusion. To minimize the hypertensive stress response associated with intubation and surgical incision, we used remifentanil (0.05-0.2 mcg/kg/min) until the baby was delivered. Remifentanil was chosen because it is easily titratable with a short duration of action. The high-dose opioids were avoided to minimize neonatal adverse effects. Haas et. al reported that the use of a high-dose opioid technique with sufentanil successfully blunted the stress response to induction; however, the neonate presented with an initial Apgar score of three and required immediate intubation for respiratory depression [10]. In our case, the mother remained hemodynamically stable following induction with a MAP greater than 60 mmHg. The baby was delivered within three minutes of anesthesia induction.

**Figure 2:**
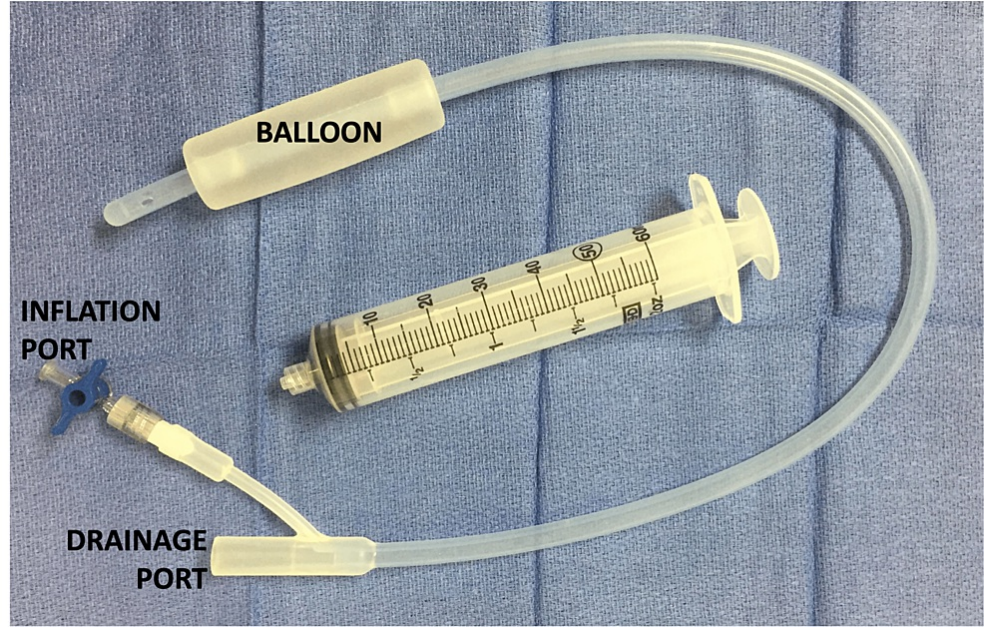
Bakri balloon designed to create an artificial tamponade to decrease uterine blood loss

Another challenge encountered during this case was the management of persistent uterine bleeding. Adequate hemostasis was obtained at the end of the Cesarean section. Therefore, the decision was made to proceed with aortic dissection repair immediately after, without performing a hysterectomy. It was in the best interest of the patient to perform aortic dissection repair without any delay to prevent further dissection progression or rupture, which could cause catastrophic sequela. A Bakri balloon was applied for anticipated uterine bleeding in the setting of full heparinization. A Bakri balloon (Figure 2) is designed to create an artificial tamponade and its ability to reduce postpartum hemorrhage has been well-demonstrated [11]. However, despite our best efforts with administrating multiple uterotonic agents, the patient lost approximately 2,000 mL of blood secondary to uterine bleeding during CPB in the setting of full heparinization and required multiple units of blood products, which made the hemodynamic management challenging. One option to consider in future cases is to perform a hysterectomy prior to dissection repair even when hemostasis is achieved at the end of Cesarean section. This would eliminate the risk of uterine bleeding during CPB. Another option that was not discussed at the time was to prevent massive bleeding involving interventional radiology to occlude or embolize uterine arteries during cardiac surgery. This technique is useful in patients with a high risk of postpartum hemorrhage [12]. However, it may not have been possible or reasonable to transport the patient emergently to the interventional radiology suite in the setting of CPB.

## Conclusions

In conclusion, the anesthetic management of pregnant patients with aortic dissection is complex and carries a high risk of morbidity and mortality. Currently, there are no official guidelines for managing pregnant patients undergoing aortic dissection repair and the literature review is limited to case reports. Each case requires an individualized multidisciplinary discussion to determine the ideal management for both the mother and fetus based on the patient’s presentation and the available resources at the institution. Our patient underwent a Cesarean section followed immediately by aortic dissection repair under general anesthesia. Our major intraoperative complication was high uterine blood loss in the setting of full heparinization. Performing a hysterectomy prior to CPB is a potential option to control the bleeding. Both mother and child survived without major complications.
